# Coronoidectomy for Alleviating Restricted Mouth Opening in Masticatory Muscle Tendon-Aponeurosis Hyperplasia: Integration With Genetic Mutation Analysis

**DOI:** 10.1155/crid/5591642

**Published:** 2025-06-13

**Authors:** Hongrong Zhang, Weihong Wang, Liang Wen

**Affiliations:** ^1^Department of Oral and Maxillofacial Surgery, Affiliated Stomatology Hospital of Kunming Medical University, Kunming, China; ^2^Yunnan Key Laboratory of Stomatology, Kunming, China; ^3^Department of Medical Imaging, First Affiliated Hospital of Kunming Medical University, Kunming, China

**Keywords:** coronoid process hyperplasia, masticatory muscle tendon-aponeurosis hyperplasia, restricted mouth opening, *SYNE1* gene

## Abstract

Masticatory muscle tendon-aponeurosis hyperplasia (MMTAH) is often misdiagnosed as temporomandibular joint disorders, hypertrophic masticatory muscles, or congenital maxillomandibular dysplasia due to overlapping clinical manifestations. This case study is aimed at elucidating a novel genetic association in MMTAH by reporting a patient with pathognomonic features, including chronic limited mouth opening, bilateral coronoid process elongation, tendon hyperplasia in the masseter and temporalis muscles, and concomitant fatty degeneration. Crucially, whole-exome sequencing of peripheral blood identified a heterozygous SYNE1 missense mutation (NM_182961.4:c.26359A>G, p.Met8787Val) in the proband. This variant, located in Exon 146 (Chr6:152122471), is an unreported variant at this locus and predicted pathogenic by in silico tools, suggesting its potential role in MMTAH pathogenesis. The study highlights the importance of genetic screening in atypical presentations to refine diagnosis and understand disease etiology.

## 1. Introduction

Masticatory muscle tendon-aponeurosis hyperplasia (MMTAH), a new disease entity, is associated with abnormal hyperplasia of the masseter and temporal tendons as well as the bilateral tendon-aponeurosis [1–4]. The clinical manifestation of this condition is marked by a distinctive square-shaped mandibular angle. Most patients experience a noticeable restriction in mouth opening during adolescence, and this challenge tends to escalate with age. It may also be accompanied by pain during chewing. MMTAH is commonly misdiagnosed or inadequately diagnosed due to its rarity in clinical practice and the little knowledge of its pathophysiology. The current study presents a typical MMTAH patient with classical clinical and imaging features. To further explore the potential genetic basis of MMTAH, we performed whole-exome sequencing (WES) in this case, aiming to identify disease-associated variants that may contribute to its pathogenesis. Our findings provide new insights into the molecular mechanisms of MMTAH and may serve as a theoretical basis for its clinical diagnosis.

## 2. Case Presentation of Clinical Data

**Figure 1:**
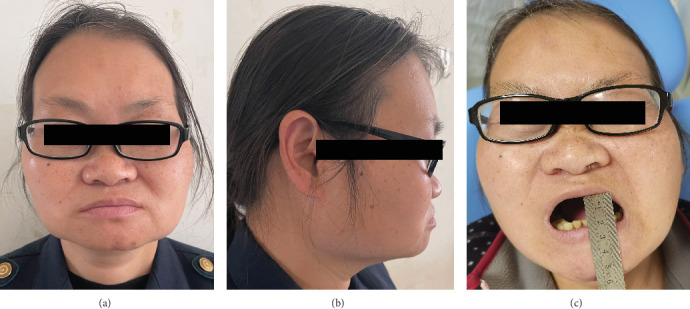
Facial morphology of patients with MMTAH. (a, b) The MMTAH patient presents with a square-shaped facial appearance. (c) The patient experiences limited mouth opening, with an interincisal distance of only 1.5 cm.

A 47-year-old female patient presenting with restricted mouth opening for more than 10 years was admitted into the Department of Oral and Maxillofacial Surgery of the Affiliated Stomatology Hospital of Kunming Medical University in April 2022. The patient complained of trouble chewing, painful biting, and sporadic painful discomfort near her temporomandibular joint. The patient exhibits a square-shaped facial appearance, with anterior teeth displaying edge-to-edge occlusion. Additionally, the approximate mouth opening is noted to be 1.5 cm. It is observed that during mouth opening, the mandible deviates to the left (Figure 1). Poor oral hygiene was noticeable in the mouth, which was covered in dental calculus throughout the entire dentition. Upon palpation examination, the patient demonstrated tenderness in the bilateral masseter and temporal muscles, which was notably pronounced when applying pressure to the anterior border of the mandibular ramus. This tenderness was further associated with hypertrophy of the temporal muscle.

## 3. Case Presentation of Radiographic Data

**Figure 2:**
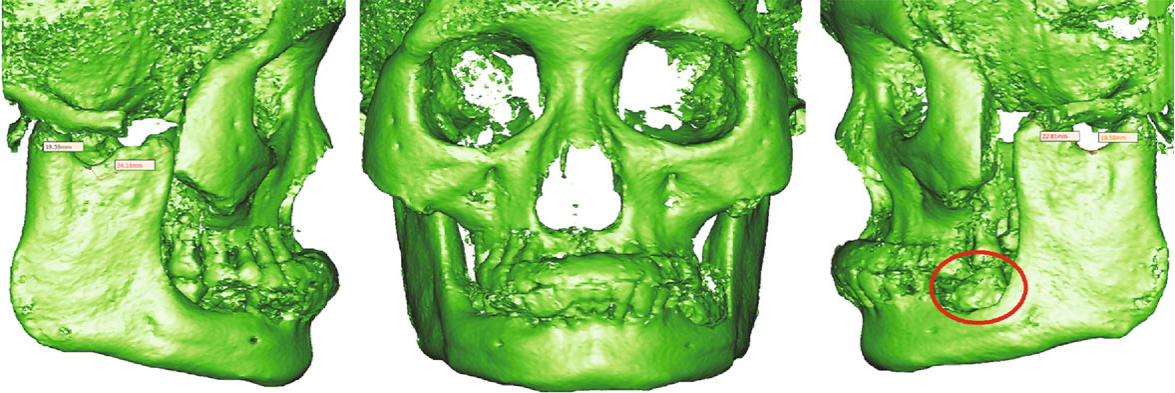
Bilateral coronoid process and condyle in patients with MMTAH: The length, thickness, and width of the bilateral coronoid process were greater than those of the ipsilateral condyle [5]. The area and extent of necrotic bone changes around the left molar marked with a red circle.

The CBCT (cone beam computed tomography) scan revealed necrotic bone development in the left mandibular molar area, bilateral irregular expansion of the coronoid process, and bilateral mandibular hypertrophy (Figure 2). CBCT data were then imported into the 3D reconstruction program SimPlant Pro 11.04 in the DICOM format; a 3D virtual model of the head was created by setting the value of thresholding in segmentation at 800–3071, and the length of the coronoid process was measured. During the measurement process, we defined two key anatomical landmarks of the mandible. The measurement of condylar length begins at the condylar vertex, which is the highest point of the condyle, and extends to the lowest point of the sigmoid notch. Similarly, the measurement of coronoid length starts at the coronoid vertex, the highest point of the coronoid process, and also extends to the lowest point of the sigmoid notch. The results showed that the length of the left and right coronoid process was longer than that of the ipsilateral condyle process (Figure 2).

**Figure 3:**
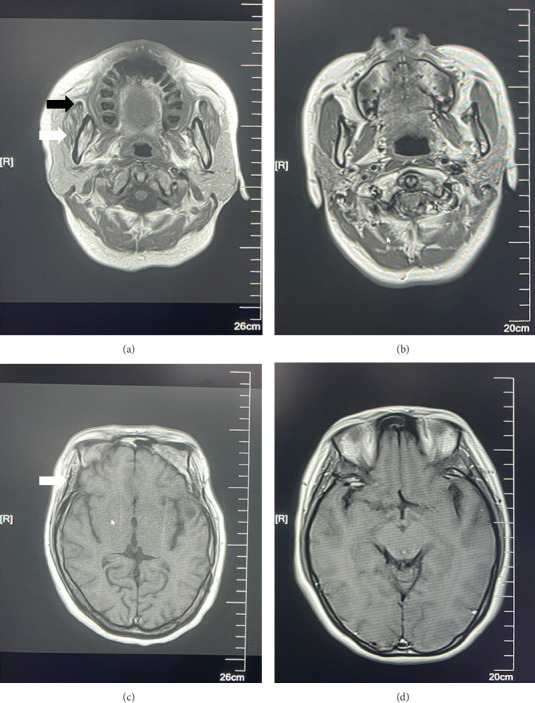
MRI scans of the masseter and temporal muscle of the MMTAH patient. (a) MMTAH masseter; (b) normal masseter muscle; (c) MMTAH temporal muscle; and (d) normal temporal muscle. The white arrows indicate muscles, and the black arrow indicates tendons. In contrast to normal, white fat infiltration and hyperplasia of the masseter tendon were observed in the temporal muscle of the MMTAH masseter.

Moreover, skull magnetic resonance imaging (MRI) examination revealed atrophy of the muscular fibers, together with white fatty granules inside the temporal and masseter muscles (Figure 3). We confirm that written informed consent was obtained from the patient for the publication of clinical data, radiographic images, and any identifiable clinical photographs.

Finally, the diagnosis of MMTAH is not only based on clinical analysis but also on imaging analysis. The diagnosis of MMTAH was established according to the following clinical criteria proposed by Yoda et al. [1–4]: (1) chronic progressive limitation of mouth opening since adolescence; (2) bilateral square mandibular angles with masseter hypertrophy; (3) tenderness on palpation of temporal/masseter muscles; (4) imaging evidence of coronoid process elongation with coronoid/condyle length ratio > 1; and (5) imaging identification of hyperplastic tendons in masticatory muscles. MMTAH should be clinically suspected when patients present with the characteristic triad of features (Criteria 1–3), with subsequent imaging confirmation required to verify the presence of osseous and tendinous abnormalities (Criteria 4 and 5).

## 4. Treatment and WES

Under general anesthesia, an intraoral approach was used to perform the bilateral coronoidectomy. The masseter tendon was substantially thickened during surgery and was continuous with the anterior border of the mandibular ramus; meanwhile, the bilateral mandibular coronoid processes were wrapped in a significant amount of hyperplastic temporal muscle and tendon-aponeurosis. Using electric knives, the temporal and masseter muscles and tendon-aponeurosis were excised, followed by the removal of bilateral coronoid processes. When the temporal tendon-aponeurosis was severed, an abnormal zipping sound was heard. Finally, the two left molars were extracted, and the necrotic bone was dissected by scraping. Drainage strips were placed and sutured. Three days after surgery, the patient started practicing regular mouth-opening exercises.

As for the temporal muscle and tendon-aponeurosis resected intraoperatively, they were fixed with 4% formaldehyde, dehydrated and embedded, and later stained with hematoxylin and eosin (HE). In addition, preoperatively, 4 mL of peripheral venous blood were collected from the patient for WES. The mutant gene was discovered through bioinformatic analysis, predicted by the software analyzing egg white function, and confirmed through Sanger sequencing. The protein function prediction software packages such as SIFT, Polyphen-2, LRT, MutationTaster, and FATHMMb were applied in predicting protein functional changes attributed to gene variants. The library was constructed by the capture and double-end (paired-end) sequencing methods using the IDT xGen Exome Research Panel. The following criteria were taken into consideration as a quality control: raw data > 10 G, average sequencing depth > 100X, and 20X coverage > 95%. All protein-coding genes were subject to a single mutation screening for copy number variants (CNVs), minor insertional deletions (InDel), and point mutations (SNV). The research protocol was reviewed in compliance with the Helsinki Declaration and approved by the Ethics Committee of the Affiliated Stomatology Hospital of Kunming Medical University (No. KYKQ2022MEC0085).

**Figure 4:**
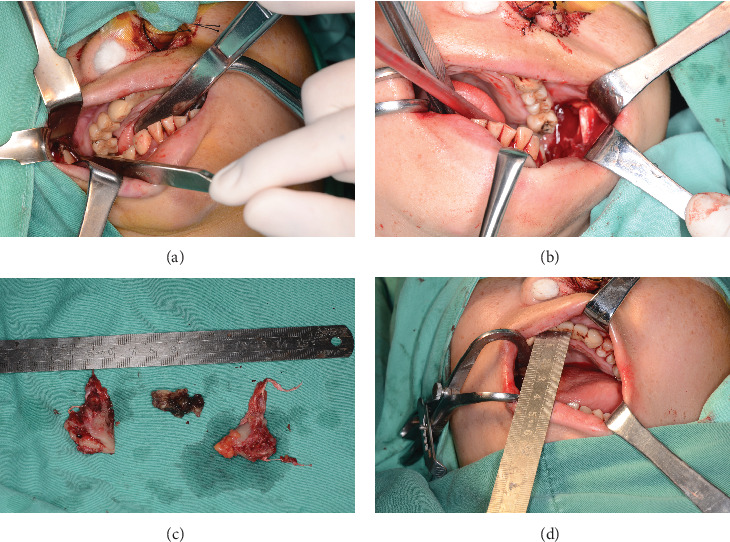
(a) Bilateral coronoid process resection was performed on the patient; (b) the coronoid process was surrounded by the proliferating tendon tissue; (c) the dead mandibular bone was removed; and (d) the intraoperative opening degree was about 4 cm.

**Figure 5:**
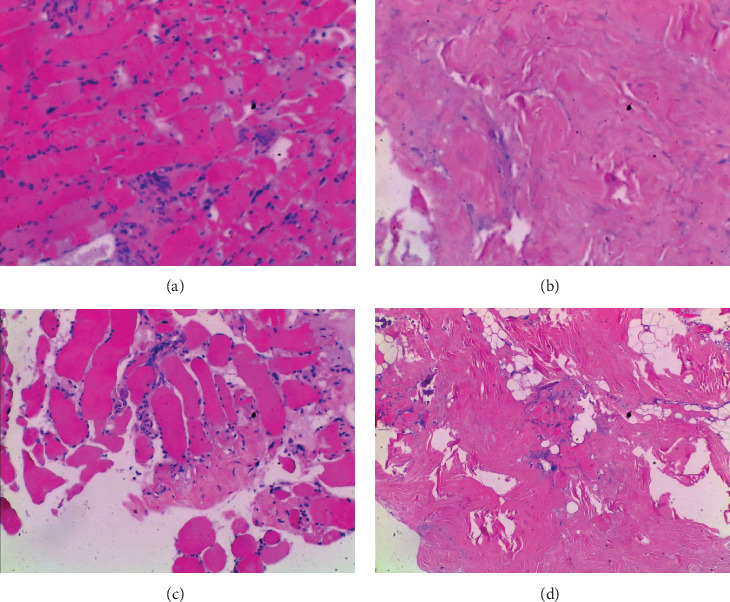
Pathological HE staining of MMTAH temporal muscle and tendon. (a) Hyperplasia of the rhabdoid tissue; (b) hyaline change; (c) focal muscle atrophy; and (d) focal tissue calcification.

**Figure 6:**
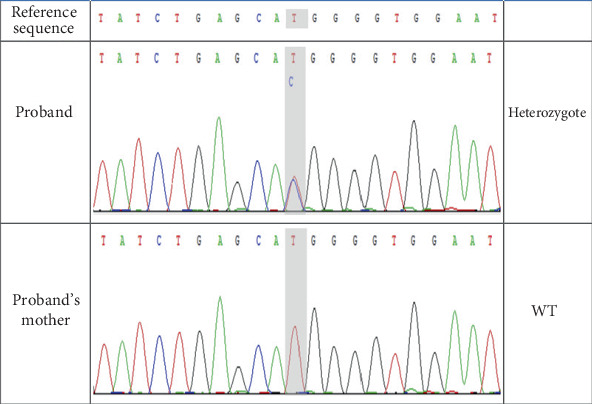
The Chr6:152122471:T>C mutation of *SYNE1* was verified by Sanger sequencing. The patient had a heterozygous mutation, and her mother was WT type. The base shown in the peak map was the reverse complementary sequence of the detected base.

Following the excision of the bilateral coronoid processes during the surgery, the patient's mouth opening increased from 1.5 to 3 cm (Figure 4). The patient recovered well postoperatively and had an opening of 3.0 cm at the 7-month follow-up. At the 37-month follow-up, the patient's mouth opening was measured at 3.0 cm, with significant improvement in masticatory muscle pain and normal occlusive function. HE staining showed hyperplasia of the temporal tendon fibrous tissue, accompanied by localized hyaline degeneration, calcification, and atrophy of the transverse muscle (Figure 5). Gene sequencing revealed a heterozygous mutation in the *SYNE1* gene (Chr6:152122471 NM_182961.4:exon146: c.26359A>G [p.Met8787Val]) of this patient (Figure 6). The software predicted that the protein function changes caused by genetic variation were mostly harmful (Table 1).

## 5. Discussion

At the 2005 meeting of the Japanese Society of Oral and Maxillofacial Surgeons, MMTAH was acknowledged as a novel disease. At the 2008 meeting of the Japanese Society of Temporomandibular Joint, the syndrome was subsequently authorized and defined [6]. The temporal tendon-aponeurosis hyperplasia, fatty degeneration of the temporal muscle, and localized foci of calcification were observed in our case of MMTAH, consistent with other literature reports [1–4, 7]. Moreover, the term “enlarged mandibular coronoid process” is defined when the thickness, length, and width of the mandibular coronoid process exceed those of the condyle, and the ratio of coronoid to condyle length surpasses 1 [8–10].

In our patient, both coronoid processes surpassed the length of their respective condyles, resulting in a diagnosis of coronoid process hyperplasia. This may be attributed to the prolonged hyperplasia of the masseter and temporal muscles, along with their associated tendon-aponeurosis complexes, leading to an abnormal increase in muscle strength. Given that the masseter and temporal muscles attach to the mandibular angle, ramus, and coronoid process, respectively, the abnormal muscle forces stimulate bone tissue growth in the direction of muscle traction. During the process of mouth opening movements, the enlarged coronoid process may interfere with the zygomatic arch, thereby restricting the extent of mouth opening.

Our prior research has additionally validated the interdependence between the hypertrophy of the mandibular coronoid process and restricted mouth opening. Surgical excision of the excessively hypertrophic coronoid process has demonstrated efficacy in improving the condition of restricted mouth opening [5, 11]. The selection of surgical approaches for coronoidectomy requires careful consideration of anatomical accessibility and aesthetic outcomes. Recent advancements in minimally invasive techniques have expanded surgical options. The intraoral approach, as utilized in our case, offers advantages including avoidance of external scarring, reduced risk of facial nerve injury, and direct visualization of the coronoid–zygomatic interface. This contrasts with traditional extracranial approaches that may compromise facial aesthetics despite providing wider exposure.

Patients with MMTAH usually experience progressive mouth opening restriction, and a tenotomy can be performed to treat MMTAH and reduce muscle tension [1–4]. As for overgrowth of the masseter tendon, Murakami et al. recommended releasing the excessive adhesion between the occlusal muscle and tendon by mandibular angle excision [12]. Otherwise, mandibular angle resection is unnecessary. However, if the patient wants to change the facial shape, surgery may be considered appropriate. In the present study, no mandibulectomy was performed according to the patient's preference. When dissecting the temporal tendon and tendon membrane intraoperatively with an electrical surgical unit, the abnormal zipping sound heard is specifically caused by calcified nodules within the hyperplastic tendon. In patients with MMTAH, elevated amounts of calcium, magnesium, and silicon are found in the tendons, as reported in previous studies [2, 7].

The pathogenic mechanism of MMTAH remains unclear. According to a few studies [1–4], it may be caused by patient-associated mutations in genes encoding proteins relevant to muscles. The expression of Myosin Heavy Chain 7 (MYH7), troponin T1 (TNNT1), Myogenic Factor 5 (MYF5), and Myogenic Factor 6 was upregulated in individuals with MMTAH, as reported by Yumoto et al. [4]. In this study, a mutation in Exon 146 of the *SYNE1* gene on Autosome 6 changed Nucleotide 26359 from adenine to guanine, which thus changed Amino Acid 8787 from methionine to valine. In normal population databases like gnomAD_exome_ALL and gnomAD_exome_EAS, this variant has not been documented, suggesting that it is a newly discovered mutation site. The *SYNE1* gene is involved in myoblast differentiation and interacts with actin via its N-terminal structural domain. This network of interactions between organelles and actin maintains subcellular spatial organization. The nesprin-1 protein, which shows high expression in skeletal and cardiac muscle, is encoded by the *SYNE1* gene. While mutations in the *SYNE1* gene may induce myogenic multiple congenital joint contractures, dilated cardiomyopathy, Emery-Dreifuss muscular dystrophy, and other muscle abnormalities [13–15].

However, the relationship between *SYNE1* mutations and the oral chewing muscles and tendons remains unclear. We speculate that *SYNE1* may activate the functions of the jaw and temporal muscles in collaboration with other genes, leading to abnormal proliferation and hyperfunction of muscles and tendons. While considering the possibility of abnormal bone development in patients, our examination of the limb bones did not reveal such phenomena. Combining our observations of increased proliferation and tension in the patient's chewing muscles, along with the attachment sites of the jaw and temporal muscles, suggests that the excessive growth of the mandibular angle and coronoid process is related to muscle traction rather than bone developmental abnormalities. Further observation and research are, of course, still needed to explore nonsurgical treatment possibilities for patients.

## 6. Conclusion

The clinical manifestations, MRI outcomes, and postoperative pathology of our patient in this study were consistent with MMTAH characteristics. WES also demonstrated a potentially pathogenic mutation site (NM_182961.4:c.26359A>G, p.Met8787Val) in the proband. This variant, located in Exon 146 (Chr6:152122471), might be closely linked to the onset of masticatory tendon hyperplasia, masticatory muscle hyperplasia, and degeneration, although its exact mechanism remains to be further investigated.

## Figures and Tables

**Table 1:** The software predicted that the protein function changes were caused by genetic variation.

**Protein function prediction platform**	***SYNE1*:exon146:c.26359A>G (p.Met8787Val)**
SIFT	Harmful
Polyphen2_HDIV	Harmful
Polyphen2_HVAR	Harmful
MutationTaster	Morbific
MutationAssessor	Moderate risk of disease
REVEL	Harmful
